# Case Report: A rare case of concurrent diabetic ketoacidosis and severe acute pancreatitis, followed by Guillain-Barré syndrome and Wernicke’s encephalopathy

**DOI:** 10.3389/fendo.2026.1923806

**Published:** 2026-08-26

**Authors:** Chanjuan Chen, Youjin Zhao, Xiyu Lin, Xiaoxin Zhang, Jia Guo, Ziqi Lin

**Affiliations:** 1 Department of Clinical Nutrition, West China Hospital, Sichuan University, Chengdu, China; 2 Department of Radiology, West China Hospital, Sichuan University, Chengdu, China; 3 West China School of Medicine, Sichuan University, Chengdu, China; 4 West China Center of Excellence for Pancreatitis, Institute of Integrated Traditional Chinese and Western Medicine, Sichuan University, Chengdu, China

**Keywords:** acute pancreatitis, case report, diabetic Ketoacidosis, Guillain-Barré syndrome, Wernicke’s encephalopathy

## Abstract

The coexistence of diabetic ketoacidosis (DKA) and severe acute pancreatitis (SAP) is a life-threatening metabolic emergency complicated by multiple organ dysfunction syndrome. The sequential development of Guillain-Barré syndrome (GBS) and Wernicke’s encephalopathy (WE) in such critically ill patients is exceedingly rare and prone to missed diagnosis. A 26-year-old obese male was admitted with unconsciousness after excessive cola ingestion. He was diagnosed with DKA, SAP, pneumonia, sepsis. During hospitalization, the patient developed progressive visual impairment, dysphagia, hoarseness, and ophthalmoplegia. Neurological examination revealed diminished to absent tendon reflexes. Typical albumin-cytologic dissociation was detected in cerebrospinal fluid. Cranial magnetic resonance imaging showed characteristic hyperintense signals in the mammillary bodies and periaqueductal region, accompanied by reduced serum vitamin B1 levels, consistent with WE. The patient was eventually diagnosed with overlapping GBS and WE. He received repeated intravenous immunoglobulin therapy and thiamine supplementation. The patient’s neurological function gradually recovered and he was discharged in stable condition. Critical illness-related systemic inflammation, metabolic derangements and infection can induce GBS, while inadequate thiamine supplementation and heightened consumption result in severe thiamine deficiency complicated by WE. Close neurological monitoring, timely thiamine supplementation, and standardized immunotherapy contribute to favorable clinical outcomes.

## Introduction

Diabetic ketoacidosis (DKA) is a life-threatening acute complication of diabetes mellitus (DM), resulting from absolute or relative insulin deficiency, which manifests as severe hyperglycemia, accelerated lipolysis, ketone body accumulation, and high anion gap metabolic acidosis (1). DKA occurs predominantly in patients with type 1 DM, it can be precipitated by infection, stress, or discontinuation of insulin therapy in those with type 2 DM. Additionally, DKA may develop in individuals with newly diagnosed or undiagnosed DM (2). Acute pancreatitis (AP), a common and potentially fatal abdominal digestive disorder, arises from the premature activation of pancreatic zymogens, leading to pancreatic autodigestion, local inflammation, systemic inflammatory response, and subsequent multiple organ injury (3). Both DKA and AP are common acute critical illnesses, with concomitant AP occurs in 10%–15% of patients with DKA (4, 5). Similarly, AP can induce acute hyperglycemia and ketogenesis via systemic stress responses and islet dysfunction, ultimately precipitating secondary DKA (6). This comorbidity mainly affects young diabetic individuals, patients with suboptimal long-term glycemic control, those on high-fat diets, excessive consumers of sugary drinks, and obese populations (4, 6). The coexistence of DKA and AP represents a severe high-risk critical condition. These two illnesses create a bidirectional vicious cycle, characterized by acute onset and rapid clinical deterioration. Patients are highly susceptible to secondary complications, including acute kidney injury, respiratory failure, sepsis, malnutrition and metabolic disturbances, as well as neurological sequelae such as Wernicke’s encephalopathy (WE). Compared with patients with isolated DKA or AP, those with DKA combined with AP exhibit significantly higher in-hospital mortality, multiorgan failure rates, ICU admission rates, and prolonged hospital stays (6, 7).

Guillain-Barré syndrome (GBS) is an acute, immune-mediated, mostly post-infectious polyradiculoneuropathy characterized by rapidly progressive, typically ascending bilateral flaccid limb weakness with reduced or absent reflexes, encompassing heterogeneous clinical and electrophysiological subtypes, associated with preceding infections via molecular mimicry in pathogenesis, manageable with intravenous immunoglobulin or plasma exchange as first-line immunotherapies, and carrying risks of respiratory failure, autonomic dysfunction, long-term disability and mortality (8, 9).

WE is an acute neuropsychiatric disorder caused by thiamine (vitamin B1) deficiency. It is characterized by the classic triad of ophthalmoplegia, mental status changes, and gait ataxia, remains highly underdiagnosed clinically, and necessitates urgent high-dose intravenous thiamine treatment (10). This disorder is most commonly observed in individuals with chronic alcohol abuse and also frequently occurs in malnourished patients with insufficient thiamine intake, impaired absorption, or increased metabolic demand (11).

We report a rare case of DKA combined with SAP complicated secondarily by GBS and WE. For clinicians, maintaining high clinical vigilance and adopting comprehensive, multidisciplinary treatment are crucial when dealing with such complex comorbid cases.

## Case description

**Figure 1:**
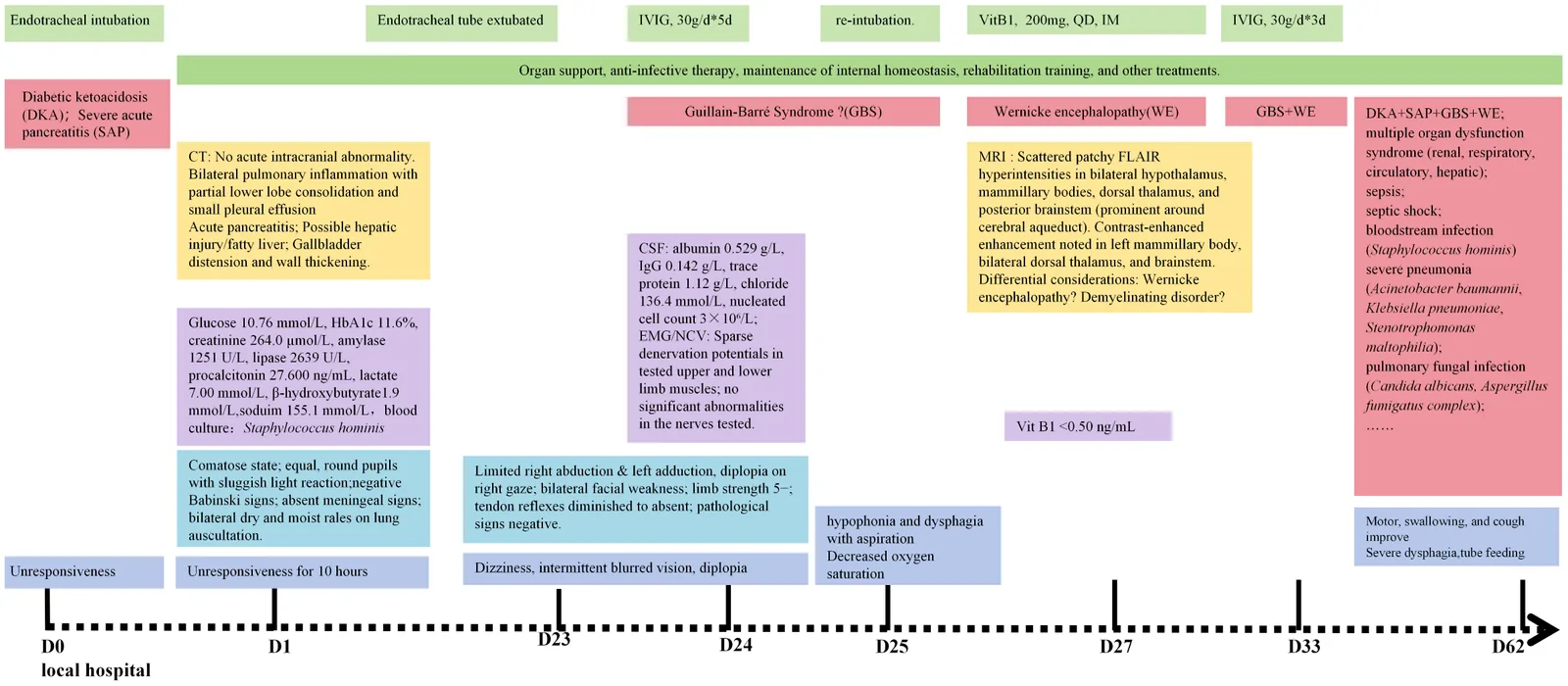
Timeline of clinical course, diagnostic workup, and therapeutic management. CT, Computed Tomography; DKA, Diabetic Ketoacidosis; GBS, Guillain-Barré Syndrome; IM, Intramuscular; IVIG, Intravenous Immunoglobulin; MRI, Magnetic Resonance Imaging; QD, Quaque Die; SAP, Severe Acute Pancreatitis; WE, Wernicke Encephalopathy.

A 26-year-old obese male collapsed at work following excessive cola consumption of 6–8 liters per day for three consecutive days. He was discovered by bystanders, who promptly called emergency services. Endotracheal intubation was performed on arrival at the local hospital. Computed tomography (CT) scan showed pancreatic enlargement, while laboratory tests confirmed severe hyperglycemia and decreased serum pH. A tentative diagnosis of diabetic ketoacidosis (DKA) combined with severe acute pancreatitis (SAP) was made. After receiving symptomatic management including glucose-lowering therapy and fluid resuscitation, the patient was transferred to our hospital 10 hours later. Review of medical history indicated an 8-year history of cholelithiasis identified on routine physical examination, and an upper respiratory tract infection three days before symptom onset. The patient denies a history of diabetes mellitus. The patient’s body mass index was 36.7 kg/m² (**Figure 1**).

On admission, the patient had a body temperature of 41°C, heart rate 165 beats/min, respiratory rate 20 breaths/min, blood pressure 144/90 mmHg, and peripheral oxygen saturation 96% (invasive ventilator in P-A/C mode, ventilator settings: FiO_2_ 60%, inspiratory pressure 16 cmH_2_O, respiratory rate 18 breaths/min, inspiratory time 1.00 s, PEEP 8 cmH_2_O). Physical examination showed the patient was unconscious. Bilateral pupils were equal, round, and sluggishly reactive to light. Pathological reflexes were absent bilaterally, and meningeal irritation signs were negative. Auscultation revealed dry and moist rales over both lung fields.

**Figure 2:**
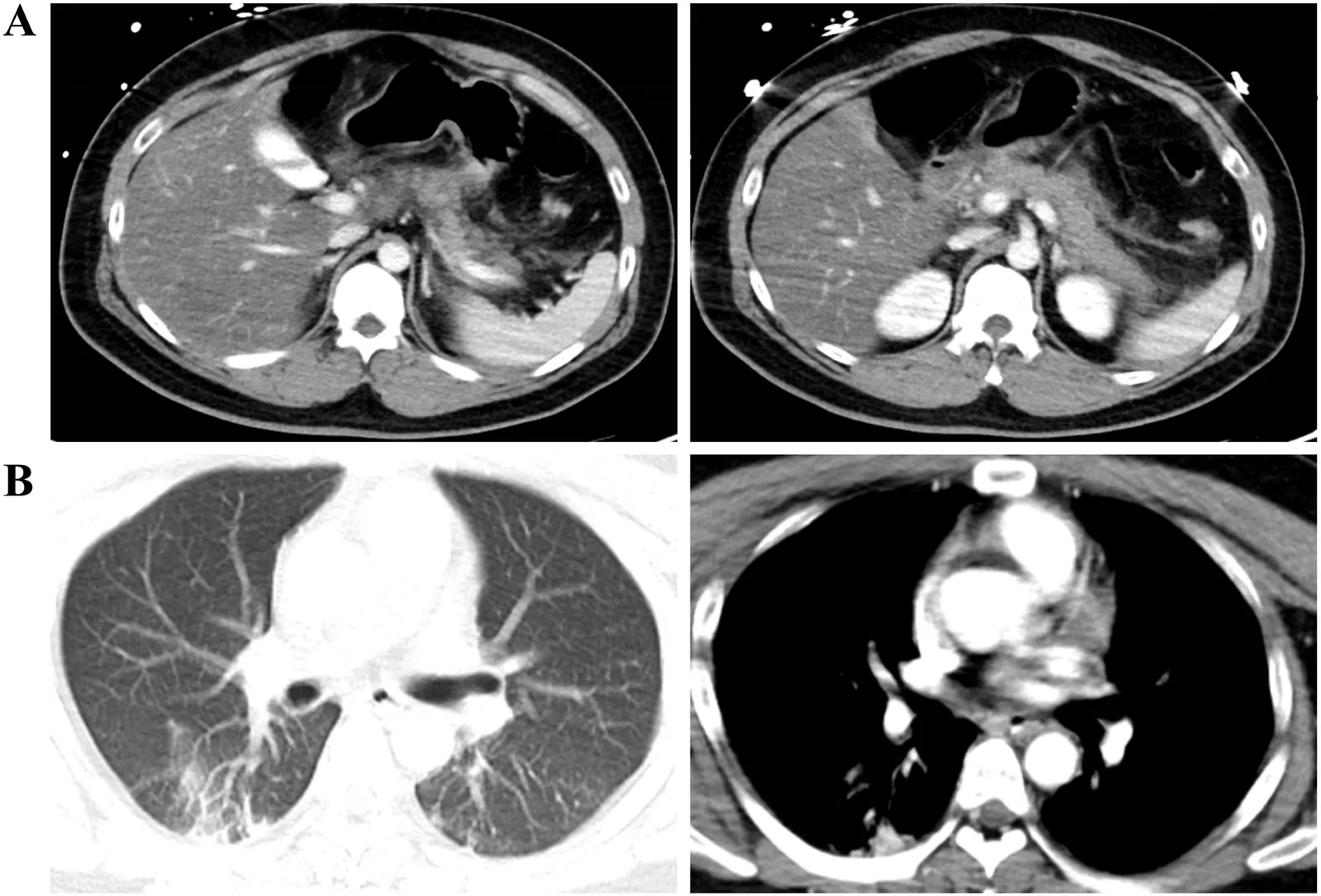
CT scan confirmed the diagnosis of acute pancreatitis and pneumonia. **(A)** Abdominal CT demonstrated pancreatic enlargement with ill-defined margins, accompanied by scattered peripancreatic exudates and fluid collections. **(B)** Chest CT shows scattered inflammatory opacities in both lungs, partial consolidation of the bilateral lower lobes, trace bilateral pleural effusion, and bilateral pleural thickening.

Laboratory tests were as follows: blood glucose 25.30 mmol/L(3.9-6.1 mmol/L), glycated hemoglobin 11.6%(4.0-6.0%); liver and renal function tests: alanine aminotransferase 2074 U/L(7–40 U/L), aspartate aminotransferase 6362 U/L(13–35 U/L), blood urea nitrogen 29.2 mmol/L(3.1-8.8 mmol/L), serum creatinine 264.0 μmol/L(68-108 μmol/L), uric acid 1499 μmol/L(202-417 μmol/L), and triglycerides 6.69 mmol/L(0.29-1.7 mmol/L); serum amylase 1251 U/L(35–135 U/L) and lipase 2639 U/L(13–60 U/L); serumβ-hydroxybutyric acid 1.9 mmol/L(≤0.28 mmol/L);complete blood count: red blood cell count 3.99 × 10¹²/L(4.30-5.80×10¹²/L), hemoglobin 124 g/L(130–175 g/L), platelet count 47 × 10^9^/L(100-300×10^9^/L), white blood cell count 10.40×10^9^/L(3.50-9.50×10^9^/L), and absolute neutrophil count 2.81×10^9^/L(1.80-6.30×10^9^/L); myocardial biomarkers: creatine kinase 725 U/L(40–200 U/L), myoglobin 3000 ng/ml(<72 ng/ml), high-sensitivity cardiac troponin T 61 ng/L(<14 ng/L), N-terminal pro-B-type natriuretic peptide 1539 ng/L (<91 ng/L); procalcitonin was 27.6 ng/mL(<0.046 ng/mL) and blood lactate 7.0 mmol/L(0.70-2.10mmol/L); blood culture confirmed *Staphylococcus hominis*. CT of the head showed no intracranial abnormalities. Chest and abdominal CT demonstrated bilateral pneumonia with partial lower lobe consolidation, a small amount of pleural effusion, acute pancreatitis (**Figure 2**), suspected hepatic injury and fatty liver changes, as well as gallbladder enlargement with thickened and rough wall.

Following admission, the patient was managed in the intensive care unit with comprehensive symptomatic and supportive therapy, including continued invasive mechanical ventilation, sedation and analgesia, fluid resuscitation, continuous intravenous insulin infusion (target blood glucose: 7.8 to 10 mmol/L), antimicrobial therapy, bedside continuous renal replacement therapy, hepatoprotective agents, expectorants nutritional support, and interventions to maintain internal homeostasis. During hospitalization, serial sputum cultures yielded *Candida albicans*, drug-susceptible *Klebsiella pneumoniae*, carbapenem-resistant *Acinetobacter baumannii*, and *Stenotrophomonas maltophilia*. After 16 days of treatment, the patient was successfully extubated and transferred to a general ward.

On hospital day 24, the patient developed dizziness, intermittent blurred vision and diplopia. Neurological assessment revealed restricted abduction of the right eye and restricted adduction of the left eye. Bilateral buccal muscle strength was reduced, while muscle strength of all four limbs was mildly decreased (grade 5^-^). Tendon reflexes were diminished to absent, with negative pathological reflexes. Emergency lumbar puncture was subsequently performed. The cerebrospinal fluid (CSF) was clear and colorless, without clot formation. CSF analysis showed a nucleated cell count of 3 × 10^6^/L (0-10× 10^6^/L) and red blood cell count of 1 × 10^6^/L (0 × 10^6^/L), with no pus cells identified. Biochemical parameters of CSF were as follows: albumin 0.529 g/L (0.14-0.23 g/L), IgG 0.142 g/L (0.010-0.030 g/L), trace protein 1.12 g/L (0.15-0.45 g/L). This finding suggests the occurrence of protein–cell dissociation. Screening for pathogens, peripheral nerve-associated antibodies, anti-Langerhans cell antibody, anti-AQP4 IgG4 and anti-MOG IgG4 all yielded negative results. Electromyography detected sporadic denervation potentials in the examined upper and lower limb muscles, whereas no abnormal changes were observed in the tested peripheral nerves. On hospital day 25, the patient experienced deteriorated visual acuity, aggravated muscle weakness, persistent diplopia, hypophonia and cough during deglutition. GBS was diagnosed, and intravenous human immunoglobulin pH4 at a dose of 0.4g/kg/day was initiated. On the same night, the patient suffered postprandial cough accompanied by a continuous decline in blood oxygen saturation, and repeat endotracheal intubation was therefore performed.

**Figure 3:**
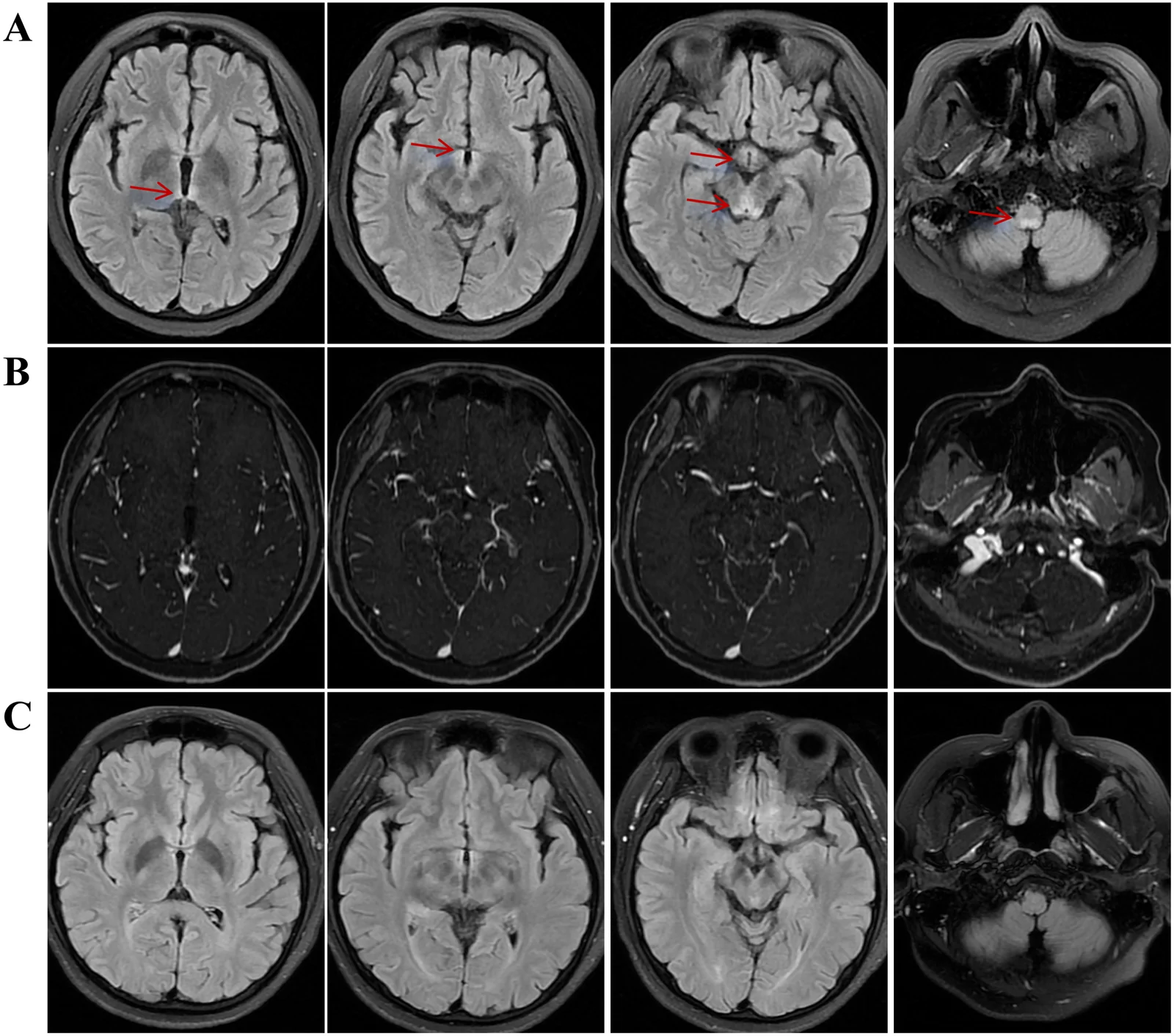
Intracranial MRI abnormalities at baseline **(A, B)** and follow-up **(C)**. **(A)** Baseline FLAIR images demonstrate scattered patchy hyperintense lesions involving the bilateral dorsal thalami, hypothalamus, mammillary bodies, and posterior brainstem, most prominently in the periaqueductal region of the midbrain (rea arrow). **(B)** Baseline contrast-enhanced images show scattered enhancing lesions in the bilateral dorsal thalami, left mammillary body, and brainstem. **(C)** Follow-up FLAIR images demonstrate near-complete resolution of the previously observed hyperintense lesions.

Subsequent cranial magnetic resonance imaging (MRI) revealed scattered, patchy, slightly hyperintense FLAIR signals in the bilateral hypothalamus, mammillary bodies, dorsal thalamus, and posterior brainstem, predominantly in the periaqueductal region. Contrast-enhanced scanning showed scattered enhancement in the left mammillary body, bilateral dorsal thalamus, and brainstem, suggestive of WE or demyelinating lesions (**Figure 3**). Laboratory tests showed vitamin B1 < 0.50 ng/mL (2.40-9.02 ng/mL), supporting the diagnosis of WE. Intramuscular vitamin B1–200 mg once daily was initiated.

Following reintubation, the patient developed recurrent high fever, and *spergillus fumigatus* was isolated from bronchoalveolar lavage fluid. The patient received intensive care, combined antifungal and antibacterial therapy, nasoenteric enteral nutrition, and supportive management to maintain internal homeostasis. After 10 days of the after mentioned comprehensive supportive therapy, the patient was successfully extubated once again. On the 33rd day of hospitalization, a second course of intravenous human immunoglobulin PH4 0.4g/kg/day was given for 3 days, combined with continuous intramuscular vitamin B1–100 mg twice daily. After treatment, the patient’s limb and swallowing functions improved, with muscle strength reaching grade 4^+^ in both upper limbs and the right lower limb, and grade 4 in the left lower limb. The patient exhibited mild dysarthria, hypophonia, and persistent choking during eating and drinking, and an indwelling nasogastric tube was maintained for feeding. During tube feeding, routine monitoring of fasting and 2-hour postprandial blood glucose showed levels fluctuating between 5 and 10 mmol/L, and no hypoglycemic agents were required. Following approximately 4 weeks of rehabilitation training, significant improvements in limb movement and expectoration were achieved; however, severe dysphagia persisted, and tube feeding was maintained. The patient was discharged in stable conditions.

### Follow-up

The patient received home-based rehabilitation after discharge and was capable of self-care. Oral feeding was resumed with adequate food intake. Although the muscle strength of the left lower extremity was mildly weakened, the patient could walk independently and climb stairs. The patient presented with hoarseness, while his thinking ability and verbal logic remained normal. He monitored blood glucose autonomously with well-controlled glycemic levels. Three months after discharge, reexamination revealed normal blood routine, liver and renal function tests. The serum triglycerides level was 2.39 mmol/L, and the serum uric acid level was 497μmol/L. The fasting blood glucose was 5.39 mmol/L, and the glycosylated hemoglobin level was 5.9%. Diabetes-related antibodies, including glutamic acid decarboxylase antibody, insulin autoantibody and islet cell antibody, were all negative. Conventional cranial MRI: several patchy slightly hyperintense FLAIR signals were noted in the bilateral thalamus and posterior brainstem, most prominently surrounding the midbrain aqueduct. Near-complete resolution of the previously observed hyperintense lesions compared with previous scans, suggestive of WE (**Figure 3C**). Chest plain CT: bilateral pulmonary inflammatory lesions were markedly reduced relative to prior imaging, with only scattered mild inflammatory foci residual.

## Discussion

We report a unique case of DKA complicated by SAP with subsequent secondary GBS and WE. This case highlights an obese young male without prior DM who initially presented with loss of consciousness consuming excessive soft drink, was subsequently diagnosed with the fatal triad of DKA, hypertriglyceridemia, and SAP that culminated in multiple organ failure and severe infection, and further developed GBS and WE, representing an exceptionally rare association scarcely documented in the literature.

The intimate bidirectional association between DKA and SAP serves as the initiating link of this complication chain. An upper respiratory tract infection presented three days before admission, and blood cultures obtained on admission confirmed sepsis. Sepsis was the presumed primary precipitant of DKA in this patient with undiagnosed diabetes. Following DKA onset, polydipsia drove excessive fluid intake, while continued consumption of high-sugar cola imposed a heavy carbohydrate load, exacerbating hyperglycemia, osmotic dehydration, and ketone accumulation. This dietary behavior served as a secondary synergistic factor of the metabolic crisis. Previous studies have shown that more than half of patients with newly diagnosed DKA have a history of excessive sugar-sweetened beverage intake prior to onset; these patients are predominantly young, obese males with no autoimmune evidence, exhibit significant recovery of islet function after treatment, and achieve considerably higher rates of 1-year diabetes remission (12, 13). Four case reports have described the occurrence of DKA-AP following excessive consumption of soft drinks (20–23), which is highly similar to our case.

Obesity, as a well-recognized independent risk factor for both metabolic disturbance (14, 15) and pancreatic injury (16), driving the occurrence and progression of both diseases via insulin resistance, chronic inflammation, lipotoxicity and hypertriglyceridemia (17, 18). Metabolic acidosis, excessive ketone body accumulation, and systemic inflammatory response induced by DKA can further damage pancreatic microcirculation, activate pancreatic digestive enzymes, and exacerbate pancreatic acinar cell necrosis. In return, SAP aggravates insulin resistance, disrupts glucose and lipid metabolism homeostasis, and thereby worsens the severity of DKA, forming a vicious cycle that elevates the risk of multiple organ dysfunction. Clinically, the gastrointestinal symptoms of DKA, including nausea and vomiting, may overlap with the abdominal manifestations of SAP (4). In addition, DKA itself can induce transient mild pancreatic enzyme elevation (19), which easily leads to missed or delayed diagnosis of pancreatic injury. Therefore, dynamic monitoring of serum amylase and lipase, as well as abdominal imaging, is essential for young obese patients with DKA, regardless of the presence of typical abdominal pain.

GBS is frequently preceded by upper respiratory tract infections or gastroenteritis. Established antecedent pathogens include *Campylobacter jejuni, Haemophilus influenzae, Mycoplasma pneumoniae*, Cytomegalovirus, Epstein-Barr virus, Zika virus, influenza virus, and Hepatitis E virus. In this case, the patient presented with an upper respiratory tract infection of unknown etiology three days prior to the onset, which was subsequently complicated by severe pulmonary and bloodstream infections after admission. Sputum cultures yielded *Acinetobacter baumannii, Klebsiella pneumoniae, Stenotrophomonas maltophilia, Candida albicans, and Aspergillus fumigatuscomplex*, while blood cultures isolated *Staphylococcus hominis*. Although these organisms are not conventionally associated with GBS, previous case reports have documented GBS following bloodstream infections caused by *Enterococcus faecalisand* and *Acinetobacter baumannii*. Additionally, DKA and SAP were identified as potential precipitating factors. Currently, there are 6 case reports describing patients with DKA who developed GBS within several days to two weeks without preceding infection, surgery, or vaccination (24–29). Only 2 case reports have documented the co-occurrence of AP and GBS (30, 31). The metabolic electrolyte disorders caused by uncontrolled DKA and systemic inflammatory storm triggered by SAP may collectively damage the blood-nerve barrier. Massive release of inflammatory cytokines induces autoimmune-mediated demyelination of peripheral nerves, ultimately resulting in acute inflammatory demyelinating polyneuropathy. GBS developed on day 24 after remarkable remission of DKA and SAP. Preceding infection followed by late-onset GBS during the recovery phase indicates that the inflammatory storm triggered by DKA and SAP may provide a susceptible background, in which antecedent infection initiates molecular mimicry and subsequent autoimmune injury, ultimately causing demyelination and axonal damage of peripheral nerves. Additionally, critical illness-related polyneuropathy secondary to SAP may overlap with GBS manifestations, increasing the difficulty of early differentiation. In this case, the progressive symmetrical limb weakness and areflexia after the remission of acute pancreatic and metabolic symptoms were highly consistent with the clinical characteristics of GBS. Lumbar puncture and electromyography can facilitate definitive diagnosis, and early administration of intravenous immunoglobulin is critical to inhibit autoimmune nerve injury and improve neurological prognosis.

WE, another severe neurological complication observed in this patient, was primarily attributed to thiamine deficiency induced by multiple factors. Poor oral intake during DKA and SAP hospitalization reduced thiamine intake, while hypermetabolic state and systemic inflammation accelerated thiamine consumption. Moreover, aggressive intravenous rehydration without timely thiamine supplementation further depleted residual thiamine reserves, disrupting cerebral glucose metabolism and causing damage to the mammillary bodies and periventricular gray matter. Clinical symptoms overlap exists between WE and GBS, including limb weakness and ophthalmoplegia. In this case, WE was confirmed only after cranial MRI, without which the diagnosis would have been easily overlooked and treatment delayed. This highlights that routine empirical thiamine prophylaxis is indispensable for critically ill patients with sustained inadequate oral intake to prevent WE.

This case highlights the clinical vulnerability of young obese patients to interconnected metabolic, inflammatory, and neurological complications. It is necessary to strengthen the health grading labeling of beverages to guide rational selection and consumption, thereby preventing adverse health consequences in young people. Obesity not only increases the susceptibility to DKA and AP but also exacerbates systemic inflammatory responses. For such critical patients, multidisciplinary collaborative management is indispensable: timely correction of metabolic acidosis and electrolyte disturbance, intensive pancreatic supportive treatment, rational immunotherapy for GBS, and standardized thiamine replacement for WE. Dynamic neurological assessment throughout hospitalization is required to identify early atypical neurological complications.

The patient initially presented with diabetic ketoacidosis (DKA) and subsequently achieved drug-free diabetes remission, which can be attributed to multiple interacting factors. The index DKA episode was precipitated by infectious stress. Resolution of infection eliminated persistent stress stimuli, and inpatient intensive insulin therapy effectively alleviated glucotoxicity, thereby providing a recovery window for functionally impaired pancreatic β-cells. The patient had baseline obesity and attained a total weight loss of 22 kg (23.4% of initial body weight) within 6 months. In line with the DiRECT trial findings and the twin-cycle hypothesis, substantial weight loss reduces ectopic fat deposition in the liver and pancreas, relieves lipotoxicity and improves insulin sensitivity, serving as the core driver of remission in obesity-associated diabetes (32). Long-term consumption of a high-fat and high-carbohydrate diet imposed sustained burden on pancreatic β-cells and formed the underlying predisposing background. Given the patient’s young age and preserved β-cell reserve, the adoption of a healthy dietary pattern after disease onset abolished persistent metabolic stress, prevented progressive deterioration of β-cell function, and consolidated the status of metabolic remission.

Several limitations of this case should be acknowledged. First, the exact molecular mechanism linking DKA-SAP to secondary GBS cannot be fully clarified due to the absence of long-term immune biomarker monitoring. Second, this is a single case report with limited sample size, which cannot establish a definite causal relationship among the four complications. Prospective large-sample studies are needed to further verify the correlation and underlying pathways. In conclusion, this rare case demonstrates a sequential cascade of DKA, SAP, GBS, and WE in an obese young male. Clinicians should maintain a high index of suspicion for secondary neurological complications in obese DKA patients complicated with pancreatitis. Early identification, comprehensive etiological analysis, and standardized multitarget intervention are pivotal to reduce mortality and improve long-term functional prognosis of such critically ill patients.

## Conclusion

We report a rare case of concurrent GBS and WE complicating DKA with SAP in a young obese male who consumed large volumes of beverages, successfully treated with multiteam collaborative management and followed by a favorable clinical prognosis. It adds to the limited literature on atypical combined neurological complications and highlights the need for heightened clinical awareness, early neuroimaging/neurophysiology, and prompt targeted therapy to optimize neurological outcomes.

## Data Availability

The raw data supporting the conclusions of this article will be made available by the authors, without undue reservation.
